# Superplasticity of Annealed H13 Steel

**DOI:** 10.3390/ma10080870

**Published:** 2017-07-28

**Authors:** Zhenxin Duan, Wen Pei, Xuebo Gong, Hua Chen

**Affiliations:** School of Materials Science and Engineering, Changchun University of Technology, Changchun 130012, China; 18844061816@163.com (Z.D.); peiwen19941102@163.com (W.P.); 15948334230@163.com (X.G.)

**Keywords:** H13 steel, high temperature tensile properties, superplasticity

## Abstract

H13 steel is a widely used hot work die material. A new type of hot working method is imperative to develop complex and precise dies. In this paper, the heat treatment of H13 steel (AISI) was carried out by annealing, the final structure is a point or spherical pearlite, and the grain size is about 30–40 μm. The tensile properties of the annealed microstructure were investigated at 650, 750, and 850 °C with the strain rates of 1 × 10^−3^ s^−1^, 5 × 10^−4^ s^−1^, and 1 × 10^−4^ s^−1^. The tensile fracture and microstructure were analyzed by SEM and HREM. The results show that the tensile samples reach superplasticity at the strain rate of 1 × 10^−4^ s^−1^ in the temperature range of 750–850 °C. When the temperature is 850 °C, the maximum elongation rate reaches 112.5%. This demonstrates the possibility of making superplastic forming molds. During the tensile process, the refined M_23_C_6_ and other high hardness carbides which are dispersed uniformly in the matrix, effectively inhibits grain growth and hinders dislocation movement, leading to the improvement of plasticity.

## 1. Introduction

H13 steel has many desirable properties, including high red hardness and thermoplasticity, low deformation during heat treatment, low thermal linear expansion, and excellent thermal crack resistance. There are many potential applications of H13 steel, in the construction industry, and new manufacturing processes are needed. At present, the main production method of H13 steel includes forging and cutting [1]. These methods consume large amounts of materials, and the cutting surface can readily produce microcracks and grinding cracks.

The formability of metal in the superplastic state is favorable, while the flow stress is small. It can avoid damage during machining and overcome all kinds of defects in the cavity of the H13 steel die. Therefore, the superplastic forming technology used on metal shows potential application in the special structure molds industry [2,3].

H13 steel is a widely used hot mold material. In order to reduce manufacturing costs and improve service life, it is imperative to develop a complex and precise die. Superplastic forming has unparalleled advantages, and some superplasticity properties of H13 steels have been reported. Previous research efforts focused on obtaining fine microstructure after quenching (grain size 8–20 μm). Zhai et al. [4] investigated H13 steel subjected to three cycles of quenching at 1000 °C; it was found to stretch at 840 °C with a strain rate of 1 × 10^−4^ s^−1^, and had a maximum elongation of 185%. Zhang et al. [5] also studied the superplastic forming of the bevel gear precision forging model cavity. A study on the superplastic forming process of H13 steel crankshaft die forging by Hu et al. [6] has also been reported.

Haghdadi N., Cizek P., and Beladi H. et al. [7] studies the softening mechanism of high temperature tensile of steel, and indicates that ferrite at high temperature showed a marked increase in the strain rate leading to a transition in the softening mechanism from CDRX towards a novel mechanism analogous to DDRX. The results of that study results inform the research of this paper.

In this paper, annealing H13 steel was used in high temperature tensile tests. The mechanical properties of H13 steel were studied, and the conditions of superplasticity and microstructure detail were observed. The fracture morphology, grain size, mechanical properties, and microstructure of H13 steel were analyzed by SEM and HREM, which provides a theoretical basis for superplastic forming of H13 steel die.

## 2. Experimental Materials and Methods

**Figure 1:**
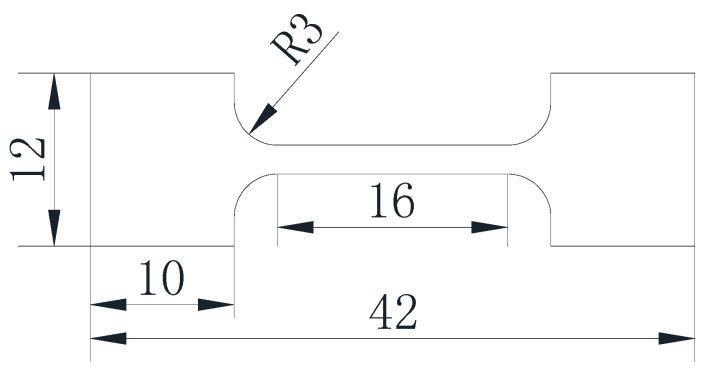
Tensile specimen size (mm).

The tensile test material is annealed H13 steel. The annealing temperature is 850 °C, the cooling rate is (10–15) °C/h, the microstructure is spherical pearlite, and the grain size is about 30–40 μm. The chemical composition (wt %): w(C) = (0.32–0.42)%, w(Cr) = (4.75–5.50)%, w(Mo) = (1.10–1.75)%, w(Si) = (0.80–1.20)%, w(V) = (0.80–1.20)%. The names and standards of the tensile test materials in different countries are shown in Table 1. The tensile specimen is made from a plate, cut from raw material steel by wire electric discharge machining [8]. Dimensions of sample as shown in Figure 1, the size of the tensile sample is designed according to the standard BS EN 10002-1 Part 1, the thickness of the sample is 2 mm. The sample surface is sanded with sandpaper, the roughness is 0.05–0.1 μm. In order to prevent oxidation during the test, the sample surface was coated with a layer of anti-oxidation coating.

Tensile specimens were tested by an electronic universal testing machine (DDL50, CIMACH, China). The experimental temperatures are 650, 750, and 850 °C, the strain rates are 1 × 10^−3^ s^−1^, 5 × 10^−4^ s^−1^, and 1 × 10^−4^ s^−1^, respectively. After completion of the tensile test, the specimens were immediately quenched, in order to maintain the morphology at a high temperature. SEM (JSM-5500LV, JEOL Ltd., Tokyo, Japan) was used to observe the macroscopic morphology of tensile fracture, and HREM (FEI Talos F20 S, FEI Technologies Inc., Beaverton, OR, USA) was used to observe and analyze the microstructure.

## 3. Experimental Results and Analysis

### 3.1. Tensile Properties Analysis

**Figure 2:**
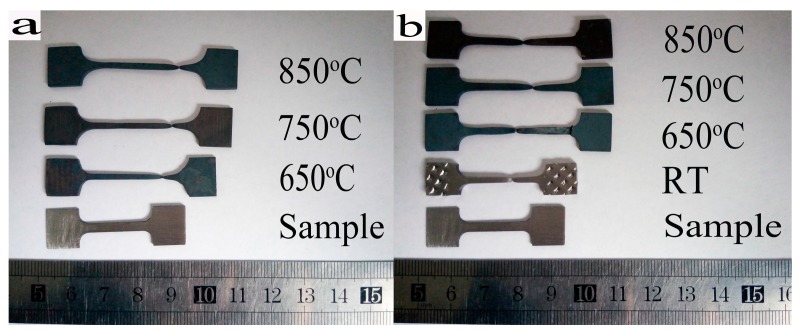
The tensile specimens with different temperature and strain rate: (**a**) 5 × 10^−4^ s^−1^; (**b**) 1 × 10^−4^ s^−1^.

The macroscopic morphology of H13 steel is before and after superplastic tensile testing. As shown in Figure 2, it reveals the plastic deformation, necking, and fracture of the tensile specimens at different temperatures and rates.

The ideal superplastic tensile test should be non-necking and has uniform deformation [9]. Due to the existence of deformation during a time-consuming tensile test, when the deformation reaches a certain stage, the internal microstructure of the material enters an unstable state as a result of the flow stress effect. Material defects such as necking occur, and the specimen begins to expand, resulting in cross-sectional contraction of the tensile specimen until fracture [10].

Figure 2a shows that tensile specimens elongate at a rate of 5 × 10^−4^ s^−1^. The elongation increases with increasing temperature, with elongation values of 82.67%, 93.75%, and 106.5%, respectively.

When the strain rate continued to decrease to 1 × 10^−4^ s^−1^, the elongation increased to 98%, 102.5%, and 112.5% with the increased temperature. The elongation was over 100% at 750 °C with this strain rate. However, the elongation of specimens at room temperature was only 32%.

With different elongation values, there are evident plastic differences in the materials under different conditions. The experimental results show that the elongation increases with the increasing temperature for a given strain rate. The elongation increases with the decrease in strain rate when temperature is held constant.

With an increase in temperature, bonds between atoms are weakened at a certain strain rate; critical shear stress thus decreases, which accelerates the grain boundary sliding and improves the elongation rate [11]. At the same temperature, the dislocation density and the propagation rate decrease with the decrease in strain rate. The point where the deformation degree of the alloy reaches maximum stress will be increased, thus enhancing elongation rate [12].

### 3.2. Tensile Stress–Strain Curve Analysis

**Figure 3:**
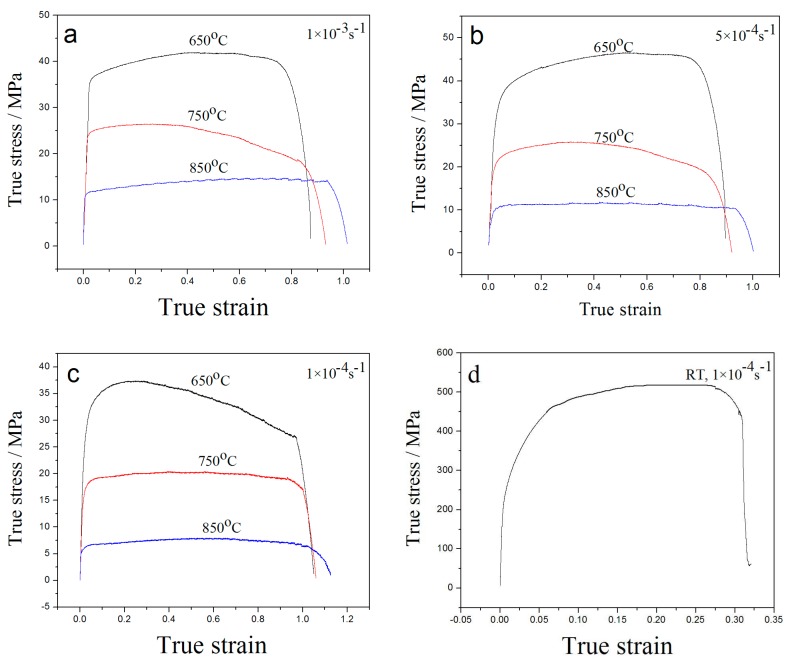
True stress–strain curve: (**a**) 1 × 10^−3^ s^−1^; (**b**) 5 × 10^−4^ s^−1^; (**c**) 1 × 10^−4^ s^−1^; (**d**) room temperature.

True stress–strain curves at three tensile temperatures and strain rates of 1 × 10^−3^ s^−1^, 5 × 10^−4^ s^−1^, and 1 × 10^−4^ s^−1^ are shown in Figure 3. It can be seen that the elastic stage appears, yet no obvious yield stage is presented. The strengthening stage and the necking stage occur in the stretching process, followed by the emergence of large plastic deformation and, finally, fracture [13].

The tensile temperature is higher than the recrystallization temperature of H13 steel. As a result, when it begins to stretch at high temperature, the tensile deformation is accompanied by the recovery and recrystallization process. Therefore, the process of hardening due to deformation, dynamic recovery, and recrystallization caused by the softening process are coexistent. Thus, the plasticity exhibited by metal at high temperature is offset by these co-existing properties. According to the true stress–strain curves shown in Figure 3, at the beginning of the cycle the hardening process dominates the softening process, so the curves show an upward trend. When the peak value is reached, the curves begin to decline and gradually become horizontal.

In contrast, when the tensile speed is greater, the result is lesser elongation, and a faster hardening rate of steel when it is under peak stress. When the strain rate is constant, the tensile strength decreases with increased temperature. At a temperature of 650 °C, the tensile strength decreases with a decrease in strain rate, while the tensile strength at 750 and 850 °C exhibits little change with the decrease of tensile rate. However, when the peak stress reaches the maximum at 850 °C, the hardening, dynamic recovery, and recrystallization processes have been kept in dynamic balance, resulting in a curve that is relatively flat until the necking occurs.

On one hand, when a tensile material is formed, it will cause sample hardening. Because of the increased dislocation and even pile up, the stress subsequently increases. On the other hand, the stress on bound atoms weakens with the temperature increase; thermal vibration amplitude and external heat addition provide thermal activation energy increases. The atomic binding capacity subsequently decreases, resulting in a reduced intensity of the dislocation barrier effect [11]. When the strain rate decreases, the deformation time increases and the plastic deformation decreases over unit time; the number of dislocations changes and the rate is reduced at the same time. This leads to lattice distortion of the internal metal becoming significantly reduced. At the same time, the slip, diffusion, and climb of dislocations are minimized. This reduces the dislocation pile up, thereby reducing the tensile strength [12].

The *m* value is an extremely important indicator of the superplastic properties of the expressed metal. For ordinary metal materials, *m* is on the order of 0.02–0.2, and the *m* of many superplastic metal materials is 0.3–0.9 [14,15].

According to the slope method, the strain rate sensitivity index *m* of H13 steel can be calculated at high temperature. A group of samples are first subjected to tensile tests at different strain rates, then true stress–strain curves are drawn. Finally, the logσ–logε curve is made by plotting the stress versus the strain rate under the same strain in the steady-state rheological stage. The slope of the curve is the *m* value.

**Figure 4:**
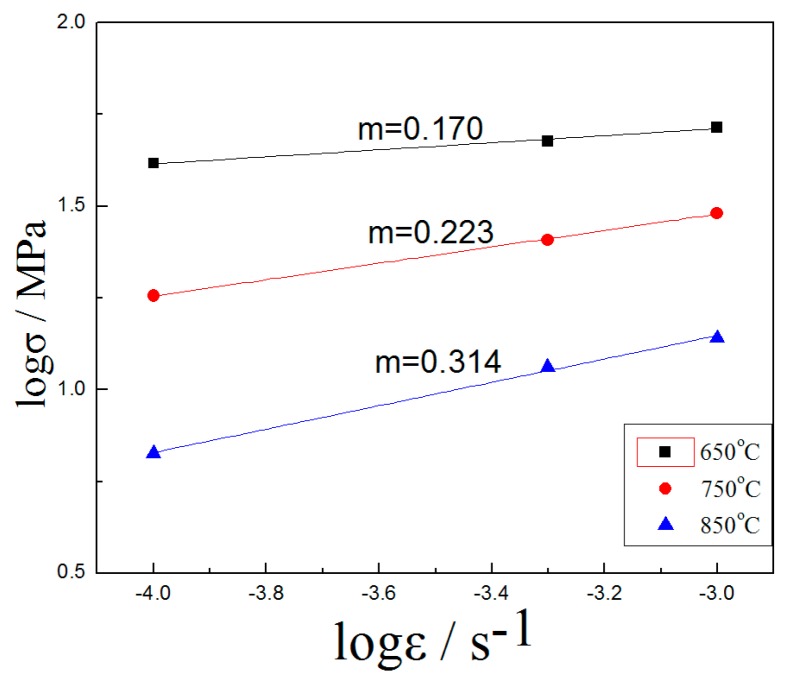
*m* Value of high temperature tensile of H13 steel.

The *m* value of the strain rate sensitivity index was 0.314 at 850 °C, as shown in Figure 4. The superplasticity of H13 steel at this temperature has been confirmed. The *m* values that did not reach the superplasticity condition were 0.223 at 750 °C and 0.170 at 650 °C.

In this experiment, H13 steel is compared with other materials in superplastic testing, Table 2. The advantages of superplastic H13 steel are presented in this experiment, including: larger grain, simple heat treatment, and less stress.

### 3.3. Microstructure and High Resolution Analysis

In order to obtain superplasticity of ordinary materials, the microstructure of the material must be stable and have equiaxed fine grains, with a grain diameter of 0.5–5 μm range. Superplasticity of the material with a diameter of more than 10 μm is difficult to achieve [4].

**Figure 5:**
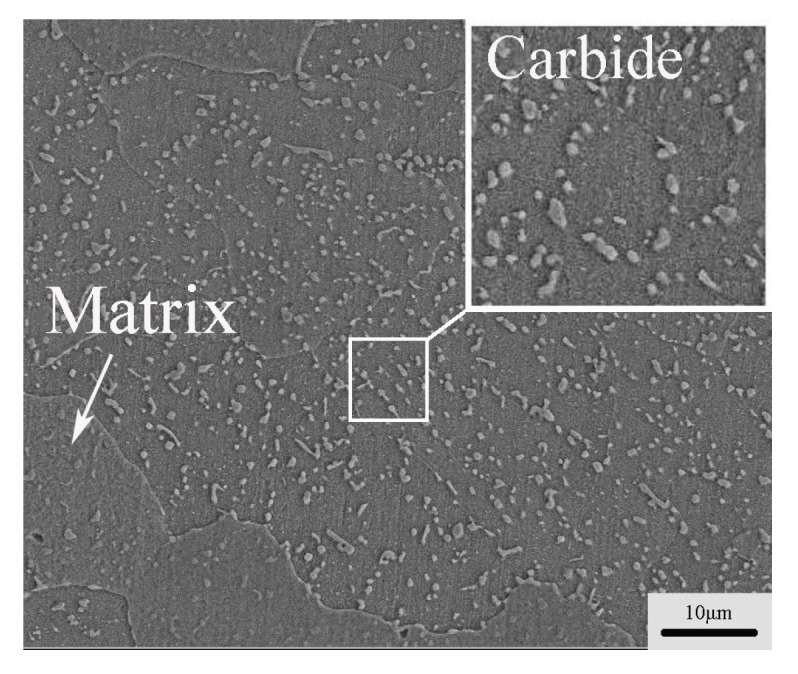
Non-stretched SEM images of H13 steel.

In the microstructure of H13 steel after annealing, there are many fine carbides distributed in the matrix, and the carbide plays an important role in the hardness and strength of the steel [21]. Fine carbides have a pinning effect on grain boundaries, whereby the grain growth is controlled effectively during the deformation process [22]. As shown in Figure 5, in the microstructure of H13 steel, the carbide phase is the size of micro refinement and dispersed homogeneously in the matrix.

**Figure 6:**
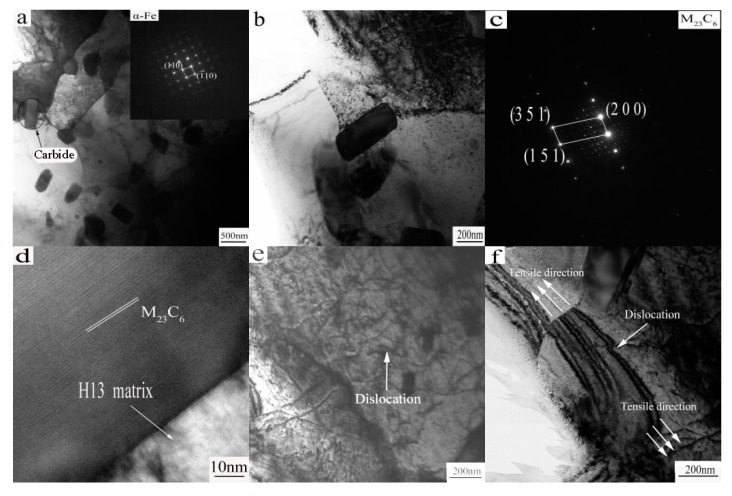
HREM morphology of the specimen after stretching: (**a**–**f**) 850 °C and 1 × 10^−^^4^ s^−^^1^.

The microstructure of superplastic tensile fracture was observed by HRTEM at 850 °C with strain rate of 1 × 10^−4^ s^−1^, as shown in Figure 6.

Figure 6a shows that there are a large number of dispersed second phase particles within the microstructure. The grain size of the ferritic matrax is 0.800–1 μm, far less than the grain size before stretching (30–40 μm). It is obvious that dynamic recrystallization occurs during the high temperature tensiling process. The dynamic recrystallization is controlled by the deformation temperature and strain rate [23,24,25], obviously, in these experimental conditions, the recrystallized grains are relatively small, which is favorable for the deformation structure to maintain high strength and toughness.

Generally, the smaller the recrystallization grain, the greater the trend of grain growth [26]. However, the fine carbides distributed in the grains and grain boundaries homogenously, where they effectively suppress the growth of recrystallized grains. Thus, the fine microstructure and superplasticity can be obtained.

The diffraction spots of the matrix are shown in the upper right corner. From the diffraction spot, it is evident that the matrix is a α-Fe phase; the enlargement of the carbide is shown in Figure 6b. Figure 6b shows that, at the grain boundaries, the carbide size is about 200 nm, and the diffraction spots are shown in Figure 6c. Figure 6d shows a high-resolution image of the carbide, the interplanar spacing is 0.66 nm, from Figure 6c,d, the carbide composition is M_23_C_6_. It is generally believed that M_23_C_6_ is one of the major carbides present in the H13 steel annealed state [26].

Figure 6e shows a high density dislocation network formed in the microstructure, as indicated by the arrow. This state indicates that the alloy plastic deformation occurred, while the previous tensile curve and elongation is very consistent. It can be inferred that dislocation takes place in the alloy during plastic deformation.

In Figure 6e, the motion of dislocations is blocked, and dislocations pile up in the vicinity of grain boundaries, resulting in the formation of a dislocation network. Dislocations interact with each other and tangle together to form dislocation cells. Dislocation cells are further converted to subgrains. Finally, subgrains grow to be small new DRX grains near grain boundaries.

As shown in Figure 6f, the dislocations and the extension of the direction of stretching under HREM are clearly shown. A large number of dislocations were caused by the formation of several high density dislocation walls in a grain. With the stretching (stretch direction as shown in arrow), the accumulation of this high density dislocation wall causes the grain to be fragmented and prompts the formation of a finer equiaxed grain, and the high density dislocation wall becomes a new grain boundary.

### 3.4. Tensile Fracture Analysis

The fiber zone, radiation zone, and shear lip zone are present in the fracture, and this is commonly called the fracture of the three elements. The crack originates in the fiber area, and then forms the radial zone by rapid expansion. When the crack spreads to the surface, a tough fracture of the shear lip forms, then finally the formation of a cup-shaped fracture is observed [27].

**Figure 7:**
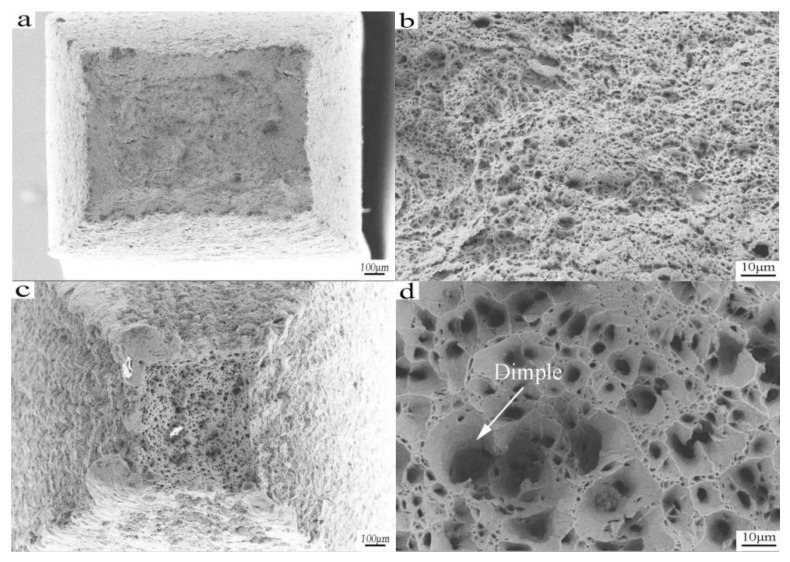
Tensile fracture morphology at room temperature and at a temperature of 850 °C and a strain rate of 1 × 10^−4^ s^−1^: (**a**,**b**) Tensile specimens at room temperature; (**c**,**d**) Tensile specimens at 850 °C.

Figure 7 shows the tensile fracture morphology at room temperature and 850 °C with a strain rate of 1 × 10^−4^ s^−1^. As shown in Figure 7a,c, the tensile specimens show obvious necking deformation; the fracture profile is square and the four pyramid fracture is evident. The fracture is initiated in the central fibrous zone, which is characterized by a fibrous undulation and roughness. The shear lip zone accounts for about four fifths of the entire section, and the distribution is symmetrical with a smooth cross section. The micro morphology of the fracture zone is equiaxed dimples (Figure 7b,d), which indicates that the fracture is a ductile fracture. Some contrast in Figure 7b,d can be noted, as the dimple is small and shallow at room temperature. However, the fracture dimple at 850 °C becomes larger, rounder, and deeper, and the fracture process presents the characteristics of micro porous polymerization.

The formation of a dimple is related to the existence of the carbide particles in the microstructure. During the stretching process, there are a large number of dislocation plugs around the second phase particles. An increase of external force and the number of dislocation pile up groups leads to stress concentration. The matrix phase of the carbide particles separates from the carbide particles. When the carbide particles are completely detached, the dimple forms. In addition, a micro crack forms in the grain boundary due to the action of external force, the phase boundary, and the location of a large number of dislocation pile ups. Due to the polymerization of the adjacent micro cracks, micro holes can be seen. Hole growth and proliferation ensues, subsequently forming connections of an instantaneous fracture [28].

As shown in Figure 7d, During the tensile process, the formed micropores are larger than at room temperature (as indicated by the arrows), which is the result of microporous polymerization. Due to the increase of micropores, the tensile strength decreases, resulting in fracturing of the tensile sample.

There is visible contrast between the samples at different temperatures. When the specimen is stretched at 850 °C, the shrinkage of the section is above 95.8%. Shrinkage is much larger than the room temperature tensile shrinkage of the section (21.6%), which is also the result of superplastic formation.

## 4. Conclusions

1. Conventional annealed H13 steel can reach superplasticity at the strain rate of 1 × 10^−4^ s^−1^ in the temperature range of 750–850 °C, the maximum elongation is 112.5% and the *m* value is 0.314.
2. The grain size of H13 steel annealed is about 30–40 μm, recrystallization occurs at high temperature tensility, and fine recrystallized grains are favorable for plastic deformation.
3. The fine carbides effectively inhibit the growth of recrystallized grains, which hinder the movement of dislocations and form the dislocation wall, a certain strength and plasticity of microstructures are kept at a high temperature and ultimately obtain superplasticity.
4. Because of micropore polymerization, the superplastic tensile fracture forms larger pores than the fracture at room temperature. With the increase of micropores, the tensile strength decreases continuously and eventually leads to fracture.

## Figures and Tables

**Table 1:** The names and standards of the tensile test materials in different countries.

Country	China	America	Germany	Japan	England
standard	GB	AISI	VDEh	JIS	BS
name	4Cr5MoSiV1	H13	X40CrMoV51	SKD61	BH13

**Table 2:** Comparison of various steel parameters [16,17,18,19,20]

Experimental Material	Treatment Process	Grain Size/μm	Maximum Elongation/%	Stretching Temperature/°C	*m*	Stress/MPa	Strain Rate/s^−1^
10CrNi5MoV	Tempered sorbite	22.5	133.5	730	_	50	6.6 × 10^−3^
10Ni3MnCuAl	850 °C three cycles of quenching	15.9	180	650	_	60	2.5 × 10^−4^
3Cr2W8V	950–1050 °C two or three cycles of quenching	2.8	228	810	_	10	1.67 × 10^−4^
W6Mo5Cr4V2	1040 °C two cycles of quenching	5.6	192	810	0.27	58.2	3.33 × 10^−4^
T10A	780 °C three cycles of quenching	4.7	415	680	0.48	10.9	2.0 × 10^−4^
H13 steel	1000 °C three cycles of quenching	4.0	185	840	0.26	31	1.6 × 10^−4^
H13 steel *	annealing	30–40	112.8	850	0.31	8.38	1.0 × 10^−4^

* The experimental steel in this paper.

## References

[1] Li Y.C., Zhao Y.L., Chen H., Liu B., Wang M.K. (2011). Forging process optimization of american standard H13 hot working die steel. Shandong Metall..

[2] Li Z.R. (1990). Principle and Application of Metal Superplastic Forming.

[3] Mukai T., Watanabe H., Higashi K. (2000). Application of superplasticity in commercial magnesium alloy for fabrication of structural components. Mater. Sci. Technol..

[4] Zhai F.B., Zhang Z.L., Xia E.H. (2001). Study on superplastic forming properties of H13 steel. Mech. Sci. Technol..

[5] Zhang Y.Z., Huang J.L., Li Y.X. (1996). Superplastic diffused welding of hot sprayed coating when superplastie forming for preeision forging dies of bevel gears. J. Luoyang Inst. Technol..

[6] Hu H., Zhang Y., Han J.C. (1994). Superplastic forming process of H13 steel forging model cavity. Met. Form. Process.

[7] Haghdadi N., Cizek P., Beladi H., Hodgson P.D. (2017). A novel high-strain-rate ferrite dynamic softening mechanism facilitated by the interphase in the austenite/ferrite microstructure. Acta Mater..

[8] Krahmer D.M., Polvorosa R., López de Lacalle L.N., Alonso-Pinillos U., Abate G., Riu F. (2016). Alternatives for specimen manufacturing in tensile testing of steel plates. Exp. Tech..

[9] Shazly M., Prakash V., Draper S. (2005). Mechanical behavior of Gamma-met PX under uniaxial loading at elevated temperatures and high strain rates. Int. J. Solids Struct..

[10] Alhussainy F., Hasan H.A., Rogic S., Sheikh M.N., Hadi M.N.S. (2016). Direct tensile testing of self-compacting concrete. Constr. Build. Mater..

[11] Darolia R., Lewandowski J.J., Liu C.T., Martin P.L., Miracle D.B., Nathal M.V. Structural Intermetallics. Proceedings of the International Symposium on Structural Intermetallics.

[12] Hu S.C., Zhang H.H., Wu X.C. (2011). Flow Stress behaviors of 30Cr3MoV steel during hot compression. Shanghai Met..

[13] Liu Z.H., Ren H.P., Li W.X. (1997). Annealing softening for H13 steel. J. Baotou Univ. Iron Steel Technol..

[14] Clemens H., Rumberg I., Schretter P. (1994). Characrerization of Ti-48Al-2Cr sheet material. Intermetallics.

[15] Padmanabhan K.A., Davies G.J. (1980). Superplasticity.

[16] Deng W.P., Wang R.F., Xue G., Yang C.F. (2014). Superplasticity of 10CrNi5MoV steel. Dev. Appl. Mater..

[17] Zhang Y.Z., Wen J.B., Chen F.X. (1995). Superplasticity and superplastic forming of steel 10Ni3MnCuAl. J. Luoyang Inst. Technol..

[18] Wang M., Yao Z.K. (1997). Study on superplasticity of W6Mo5Cr4V2 high speed steel. Forg. Stamp. Technol..

[19] Long Y.T., Xu X., Lin F.Y. (1990). Superplasticity of T10A carbon tool steel. New Technol. New Process.

[20] Yang Y.L., Liu Z.B., Kang B.X. (1987). Superplasticity of 3Cr2W8V steel and superplastic forming of mold cavity. New Technol. Process.

[21] Pogatscher S., Antrekowitsch H., Leitner H., Sologubenko A.S., Uggowitzer P.J. (2013). Influence of the thermal route on the peak-aged microstructures in an Al–Mg–Si aluminum alloy. Scr. Mater..

[22] Yamamoto Y., Brady M.P., Lu Z.P., Maziasz P.J., Liu C.T., Pint B.A., More K.L., Meyer H.M., Payzant E.A. (2007). Creep-resistant, Al2O3-forming austenitic stainless steels. Science.

[23] Shimizu I. (2008). Theories and applicabilitu of grain size piezometers: The role of dynamic recrystallization mechanisms. J. Struct. Geol..

[24] Wang Y., Shao W.Z., Zhen L., Zhang X.M. (2008). Microstructure evolution during dynamic recrystallization of hot deformed superalloy 718. Mater. Sci. Eng. A.

[25] Takaki T., Hisakuni Y., Hirouchi T., Yamanaka A., Tomita Y. (2009). Multi-phase-field simulations for dynamic recrystallization. Comput. Mater. Sci..

[26] Fu R.Y., Liu Y.K. (1992). The study of tempering and carbides of cast H13 steels. J. Met. Heat Treat..

[27] Wang S.P. (1986). Fundamentals of Metallographic Analysis.

[28] He Z.B., Fan X.B., Shao F. (2012). Formability and microstructure of AA6061 Al alloy tube for hot metal gas forming at elevated temperature. Trans. Nonferrous Met. Soc. China.

